# Effect of Ganglioside combined with pramexol in the treatment of Parkinson's disease and its effect on motor function

**DOI:** 10.5937/jomb0-42550

**Published:** 2023-08-25

**Authors:** Xinna Li, Peihai Han, Mengjiao Liu, Xiaowen Li, Shuai Xue

**Affiliations:** 1 The Affiliated Yantai Yuhuangding Hospital of Qingdao University, Department of Pathology, Yantai, China; 2 Traditional Chinese Medical Hospital of Huangdao District, Encephalopathy Department, Qingdao, China; 3 Affiliated Qingdao Central Hospital of Qingdao University, Qingdao Cancer Hospital, Department of Rehabilitation Medicine, Qingdao, China; 4 Zhangqiu District People's Hospital, Department of Endoscopy Room, Jinan, China; 5 Shandong University, Cheeloo College of Medicine, Qilu Hospital (Qingdao), Health Care Department, Qingdao, Shandong, China

**Keywords:** PD, Gangliosides, Pramipexole, Motor function, UPDRS, PD, gangliosides, pramipeksol, motorna funkcija, UPDRS

## Abstract

**Background:**

This study was aimed to evaluate the efficacy of pramipexole combined with ganglioside for PD treatment and pramipexole monotherapy, so as to provide reference for clinical practice.

**Methods:**

61 PD patients selected from June 2019 to December 2020 at our hospital were divided into two groups. The control group (n=31) was given dopasizide oral treatment, and the treatment group (n=30) was given ganglioside combined with pramipexole. The clinical efficacy, adverse reactions, motor function scores, UPDRS scores, PDQ-39 scale scores, TNF-a levels, and related serum factor levels were measured in this study.

## Abstract (sr)

**Uvod:**

Ova studija je imala za cilj da proceni efikasnost pramipeksola u kombinaciji sa gangliozidom za lečenje PD i monoterapiju pramipeksolom, kako bi se pružila referenca za kliničku praksu.

**Metode:**

61 pacijent sa PB odabran od juna 2019. do decembra 2020. u našoj bolnici podeljen je u dve grupe. Kontrolnoj grupi (n=31) je davan oralni tretman dopasizidom, a grupi za lečenje (n=30) davan je gangliozid u kombinaciji sa pramipeksolom. U ovoj studiji su mereni klinička efikasnost, neželjene reakcije, rezultati motoričke funkcije, UPDRS rezultati, rezultati na skali PDK-39, nivoi TNF-a i povezani nivoi faktora u serumu.

**Rezultati:**

U poređenju sa kontrolnom grupom, ukupna efektivna stopa je očigledno povećana. Nivoi CRP i TNF-a, ton i brzina govora, položaj sedenja i hodanja, rezultati pisanja i sposobnosti ruku su smanjeni, dok je nivo BDNF povećan u grupi na lečenju. Tokom perioda, u poređenju sa kontrolnom grupom, incidencija neželjenih reakcija u grupi koja je lečena je značajno smanjena.

**Zaključak:**

Gangliozid u kombinaciji sa pramipeksolom bio je efikasan u lečenju PD. Može efikasno smanjiti nivoe CRP i TNF-a, povećati nivo BDNF, poboljšati neurološku funkciju, poboljšati motoričku funkciju i ne povećava neželjene reakcije pacijenata. Dostojan je primene.

## Introduction

Parkinson’s disease (PD), also known as paralysis of tremor, is a degenerative neurological disease commonly seen in the middle-aged and elderly [1], clinically characterized by static tremor, motor retardation, myotonia, and postural balance disorders. If treatment is not timely, patients may lose the ability to care for themselves [2]. The incidence of PD is 1%- 2% in people in their 60s [3], but increases to 3%–4% in people in their 80s [4]. The prevalence of PD in China ranges from 16 to 440.3 per 100,000, and the annual incidence ranges from 1.5 to 8.7 per 100,000. The quality of life (QOL) of patients with PD is severely reduced, and they lack the ability to take care of themselves, which brings a heavy burden to their families [1]. Currently, drug treatment programs for PD shows a degree of efficacy. Therefore, choosing an appropriate treatment plan is essential for relieving symptoms and improving the patients’ quality life.

Levodopa has been reported to become the most effective drug for the treatment of PD. However, it has now been confirmed that most patients receiving levodopa treatment in the early stage of PD, especially those with high doses, will have motor complications. Pramipexole is a non-ergot alkaloid dopamine receptor agonist with high selectivity for the D2 subfamily of dopamine receptors and a preferential affinity for D3 receptors [5]. It is used in the single and adjuvant therapy of PD and has been approved in the USA for the treatment of early and late PD [6] [7]. Pramipexole delays motor complications caused by levodopa in the early stage of PD through a neuroprotective effect [8] [9], controls motor symptoms and alleviates depression in PD patients [10] [11]. Ganglioside, one of the major cerebral gangliosides, is a sphingolipid composed of three structural units [12]. This molecule has been regarded as an important regulator of various brain functions because of its regulation of neuronal plasticity, neuronutrient release, neurotransmission, and its interaction with neuroregulatory proteins [13]. In addition, exogenous gangliosides have been shown to affect the survival of dopaminergic neurons, glutamate neurons and cholinergic neurons [14]. The therapeutic effect of GM1 ganglioside has been demonstrated in PD patients [15] [16] and MPTP-treated mice [17] [18], showing neuroprotective or neurorepair effects [19].

Although the efficacy of pramipexole alone in PD treatment has been extensively studied, the effect of pramipexole and ganglioside combination therapy on inflammatory cytokines and on patients’ motor function has not been reported. Therefore, this study was aimed to evaluate the efficacy of pramipexole combined with gangliosides for PD treatment and pramipexole monotherapy, so as to provide reference for clinical practice.

## Materials and methods

### Research object

Total of 61 PD patients were selected from June 2019 to December 2020 at above hospital, and divided into control group (n=31) and treatment group (n=30). Inclusion criteria: According to the diagnostic criteria in the Chinese Parkinson’s Disease Treatment Guidelines (Second Edition), patients aged 50–80 years old, with an education level of elementary school or above, and who meet the diagnostic criteria for PD were collected. Exclusion criteria: Psychiatric patients; patients with poor treatment compliance; patients with heart, liver, and kidney dysfunction; patients with secondary Parkinson’s syndrome caused by poisoning, trauma, etc.; patients with oral uric acid-lowering drugs, patients with a history of gout or hyperuricemia; those with obvious allergies or adverse reactions to the drugs in this study; those with incomplete data. All patients have signed informed consent and this study was approved by the ethics committee of this hospital (Approval no. 20190436).

### Treatment methods

(1) The control group was given dopasserzide tablets (Shanghai Fuda Pharmaceutical Co., Ltd., National Medicine Zhunzi H20143411, 0.125 g/tablet, Shanghai, China) treatment, the initial dose was 0.125 g/time, 3 times/d, according to the patient’s specific conditions. The dosage can be adjusted reasonably for the condition of the disease, and the dosage can be adjusted every 2 weeks, and the maximum dosage should not exceed 0.25 g/time.

(2) The treatment group was given ganglioside combined with pramipexole: monosialotetrahexose ganglioside sodium injection (Harbin Medical University Pharmaceutical Co., Ltd., National Medicine Standard H20064601, 2 mL: 20 mg) 40 mg dissolved in 250 mL 0.9% sodium chloride injection for intravenous drip, 2 times a day; oral pramipexole hydrochloride tablets (Boehringer Ingelheim Pharmaceutical Co., Ltd., Germany, National Medicine Standard H20140917, 0.25 mg/tablet) 0.25 g/time, 3 times/d. Both groups of patients took 2 weeks as a course of treatment. The drug was stopped for 10 days after 1 course of treatment, and then the next course of treatment was continued for 3 months. During treatment, the patient’s condition was monitored, and the treatment was stop in time and make corresponding adjustments in case of abnormalities.

### Evaluation criteria

(1) UPDRS reduction rate was applied to evaluate the clinical efficacy after treatment for 4, 8, and 12 weeks [20]: including mental, behavioral and emotional, activities of daily living and motor symptoms. The higher the score, the more severe the illness.

(2) The PDQ-39 was applied to evaluate the QOL before and after treatment, including daily living activities, cognition, activities, communication, and social support. The higher the scores, the lower the QOL [21].

(3) Parkinson’s motor function score was applied to assess the motor function of the two groups after treatment for 12 weeks. It was divided into three aspects: speech intonation and speed, sitting and walking posture, writing and hands-on ability. The higher the scores, the worse the motor function.

(4) Measurement of the treatment effects: markedly effective means that the clinical symptoms disappear and vital signs return to normal, and the UPDRS score reduction rate is 30%; effective is the improvement of clinical symptoms and vital signs, 5% UPDRS score reduction rate is less than 30%; invalid is did not meet the above standards.

(5) Measurement of the incidence of adverse reactions, including insomnia, dizziness, nausea, and vomiting.

### Observation indicators

5 mL of venous blood was collected from the patient in the morning on an empty stomach, and then froze in 80°C refrigerator. ELISA kit was used to determine the serum TNF-α, serum CRP and BDNF levels, and an automatic biochemical analyzer was applied for measuring serum cystatin C.

### Statistical analysis

The data obtained was analyzed by Statistical Product and Service Solutions (SPSS) 22.0 software (IBM, NY, USA). The measurement data were expressed as (FORMULA±s) and the count data were tested by chi-square test. After the normality test, the t-test was used for those with normal distribution and the variance was uniform, the t-test was used for the non-uniform variances, and the non-parametric test was used for the non-normal distribution. P<0.05 was considered as statistical difference.

## Results

### Measurement of baseline data

Treatment group: 30 males and 17 females; age 46–73 years, average age (59.83±4.15) years; course of disease 1–8 years, average course of disease (4.13±0.45) years; according to Hoehn-Yahr classification: grade I (6 cases), grade II (8 cases), and grade III (16 cases). Control group: 43–74 years old, average age (58.96±4.76) years; disease course 1–7 years, average disease course (3.97±0.71) years; According to Hoehn-Yahr classification: grade I (7 cases), grade II (9 cases), and grade III (15 cases). The two groups of general data are comparable (P>0.05) (Table 1, Table 2), indicating that the two groups of subjects had good consistency and comparability when they were enrolled in the group.

**Table 1:** General characteristics of the two group.

Feature	Control	Treatment
Age		
<60	18(58.1)	18(60.0)
≥60	13(41.9)	12(40.0)
Average age	59.83±4.15	58.96±4.76
Gender		
Male	21(67.7)	22(73.3)
Female	10(32.3)	8(26.7)
Exercise habits		
Yes	8(25.8)	10(33.3)
No	23(74.2)	20(66.7)
Weight		
<55	11(35.5)	9(30.0)
≥55	20(64.5)	21(70.0)
Course of disease	4.13±0.45	3.97±0.71

**Table 2:** Hoehn-Yahr classification of the two groups.

Group	Grade I	Grade II	Grade III
Control group	7(22.6)	9(29.0)	15(48.4)
Treatment group	6(20.0)	8(26.7)	16(53.3)

### Measurement of UPDRS score changes

**Figure 1:**
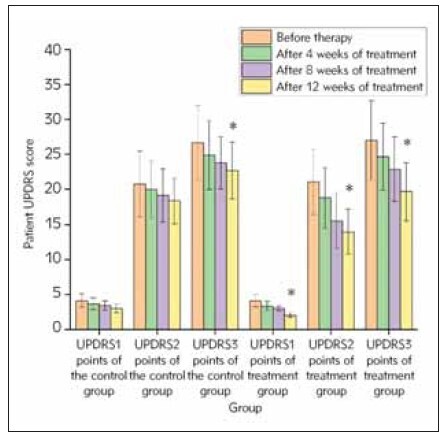
Measurement of UPDRS scores of patients after treatment. *P<0.05 vs before treatment

The difference of UPDRS1, UPDRS2, and UPDRS3 scores of the two groups was not statistically significant before treatment. After treatment for 4, 8, 12 weeks, the UPDRS scores were obviously decreased in the two group, and the UPDRS scores in the treatment group were reduced markedly in comparison with control group after treatment for 12 weeks, as shown in Figure 1, suggesting gangliosides combined with pramipexole and dopasazine tablets can better improve the patient’s motor function, balance ability and daily activity ability.

### Measurement of PDQ-39 scale points

The PDQ-39 scores of patients in the two group did not change significantly before treatment. After 12 weeks of treatment, the PDQ-39 scores of the treatment group were significantly reduced in comparison with control group, as shown in Table 3.

**Table 3:** Measurement of PDQ-39 scores after treatment. *P<0.05 vs. the control group; #P<0.05 vs. before treatment

Group	Before treatment	After treatment
Control group	48.32±6.73	41.64±4.68*
Treatment group	49.13±6.82	37.23±4.03*#

### Parkinson’s motor function score

After treatment, the scores of speech, tone and speed, sitting and walking posture, writing and hand ability in the two groups were reduced. The motor function score in the treatment group was markedly reduced versus to control group, indicating that the degree of improvement of the motor function was better after treatment (Table 4).

**Table 4:** Measurement of motor function scores. *P<0.05 vs. the control group; #P<0.05 vs. before treatment

Time	Group	Speech intonation <br>and speed	Sitting and walking <br>posture	Writing and <br>hands-on skills
Before treatment	Control group	11.17±2.45	8.71±2.12	4.47±1.05
	Treatment group	11.32±2.03	8.67±1.84	4.52±1.19
After treatment	Control group	8.97±1.41*	6.45±1.56*	2.74±0.41*
	Treatment group	6.03±1.25*#	4.23±1.47*#	1.11±0.39*#

### Measurement of treatment effects

**Figure 2:**
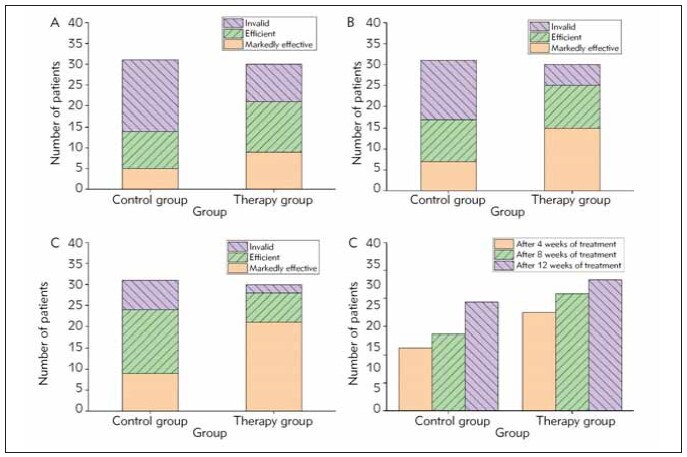
Comparison of treatment effects after treatment. (A) Comparison of clinical efficacy at 4 weeks after treatment, (B) Comparison of clinical efficacy at 8 weeks after treatment, (C) Comparison of clinical efficacy at 12 weeks after treatment, (D) Comparison of total effective rate after treatment.

The total effective rate in the treatment group was elevated obviously versus to control group after treatment for 4, 8 and 12 weeks, as shown in Figure 2.

### Measurement of adverse reactions

After treatment, the incidence of vomiting, depression and anorexia in the treatment group were obviously decreased. After targeted treatment, the patients recovered well without causing more serious consequences. The incidence of palpitations, nausea, and diarrhea in the treatment group was reduced, but there was no statistical difference, as shown in Table 5.

**Table 5:** Proportion of adverse reactions. *P<0.05 vs. the control group.

Adverse reactions	Control group <br>(n=31)	Treatment group <br>(n=30)
Anorexia	6 (19.4)	3 (10.0)
Headache	4 (12.9)	1 (3.3)
Vomit	3 (9.7)	2 (6.7)
Nausea	3 (9.7)	2 (6.7)
Listless	3 (9.7)	1 (3.3)
Diarrhea	4 (12.9)	2 (6.7)
Liver damage	2 (6.4)	1 (3.3)
Kidney damage	3 (9.7)	2 (6.7)
Total incidence of <br>adverse reactions	17 (54.8)	10 (33.3)*

### Measurement of TNF-α levels after treatment

**Figure 3:**
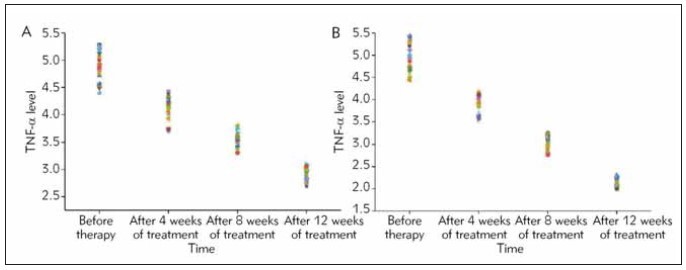
(A) Measurement of serum TNF-α levels after treatment in Control group and (B) Treatment group.

The serum TNF-α levels of the two groups before treatment were (4.87±0.41) ×10^-3^ mg/L and (4.94±0.50) ×10^-3^ mg/L, respectively, and there was no significant difference, indicating that they were comparable. The TNF-α level continued to decrease with the increase in the course of treatment. The treatment group was treated after 8 weeks, there was a statistical difference compared with before treatment. After 12 treatment, both groups were significantly reduced (Figure 3). Importantly, the TNF-α level in the treatment group was obviously down-regulated, relative to control group.

### Measurement of related serum factors

In the treatment group, the serum BDNF was (11.23±2.44) ×10^-3^ mg/L, CRP was (5.98±1.71) ×10^3^ mg/L, and Cystatin C was (1.08±0.32) ×10^3^ mg/L; the control group was (11.41±2.32) ×10^-3^ mg/L, (5.96±1.66) ×10^3^ mg/L, (1.08±0.27) ×10^3^ mg/L, respectively, and there was no significant difference, and they were comparable before treatment. After treatment for 4 weeks, the control group’s serum BDNF was (12.53±2.42) ×10^-3^ mg/L, CRP was (5.41±1.44) ×10^3^ mg/L, and Cystatin C was (1.08±0.24) ×10^3^ mg/L, the treatment group was (14.22) ±2.77) ×10^-3^ mg/L, (4.98±1.47) ×10^3^ mg/L, (1.04±0.27) ×10^3^ mg/L. After treatment for 8 weeks, the treatment group’ serum BDNF was (16.03±3.10) ×10^-3^ mg/L, CRP was (4.10±1.22) ×10^3^ mg/L, and Cystatin C was (1.01±0.15) ×10^3^ mg/L; the control group was (13.97±2.) ×10^-3^ mg/L, (5.03±1.21) ×10^3^ mg/L, (1.07±0.18) ×10^3^ mg/L, respectively. After treatment for 12 weeks, the treatment group’ serum BDNF was (18.55±3.47) ×10^-3^ mg/L, CRP was (3.47±1.04) ×10^3^ mg/L, and Cystatin C was (0.97±0.11) ×10^3^ mg/L, the control group was (14.79) ±2.93) ×10^-3^ mg/L, (4.59± 1.17) ×10^3^ mg/L, (1.06±0.16) ×10^3^ mg/L, there was statistically significant difference, as shown in Table 6.

**Table 6:** Measurement of serum BDNF, serum cystatin C and serum CRP levels after treatment. *P<0.05 vs. control group; #P<0.05 vs. before treatment

Group		Before <br>treatment	After 4 weeks <br>of treatment	After 8 weeks <br>of treatment	After 12 weeks <br>of treatment
Control group	BDNF <br>(×10^-3^ mg/L)	11.41±2.32	12.53±2.42	13.97±2.75	14.79±2.93#
	CRP <br>(×10^3^ mg/L)	5.96±1.66	5.41±1.44	5.03±1.21	4.59±1.17#
	Cystatin C <br>(×10^3^ mg/L)	1.08±0.27	1.08±0.24	1.07±0.18	1.06±0.16
Treatment group	BDNF <br>(×10^-3^ mg/L)	11.23±2.44	14.22±2.77	16.03±3.10*	18.55±3.47*#
	CRP <br>(×10^3^ mg/L)	5.98±1.71	4.98±1.47	4.10±1.22*	3.47±1.04*#
	Cystatin C <br>(×10^3^ mg/L)	1.08±0.32	1.04±0.27	1.01±0.15	0.97±0.11

## Discussion

Clinically, dopasizide tablets are the first choice as a treatment drug for PD patients, which can effectively improve the symptoms of PD patients. However, after 3 to 5 years of use of the drug, its efficacy gradually diminished, and it also increased adverse drug reactions [22]. Pramipexole, a dopamine receptor agonist, relieves the motor symptoms of PD patients. It has obvious advantages in improving depression in patients [23], which can be activated by Dopamine receptors in the substantia nigra and striatum of the midbrain maintain the normal discharge of striatal neurons [24], and can also protect dopamine cells and reduce nerve cell damage or death [25] [26] [27]. Therefore, the combination of pramipexole and dopaserizide can not only reduce the dose of dopaserizide, but also significantly improve the motor function of PD patients.

Dopaserizide is a compound preparation composed of benserazide and levodopa, which has a decarboxylation effect. As an intermediate in the biosynthesis of dopa gum, levodopa can effectively treat tremor paralysis and relieve PD patients’ symptoms. However, with the prolongation of treatment time, dopasrazide is likely to cause »end-of-dose phenomenon «, and a single drug cannot achieve the best effect. However, clinical trials have proved that the new dopamine receptor agonist pramipexole hydrochloride tablets have a significant effect on the motor symptoms and non-motor symptoms of Parkinson’s [28]. This study displayed that PD patients treatment with pramipexole combined with ganglioside showed a significant increase in UPDRS1, UPDRS2, UPDRS3 and PDQ-39 scores. Importantly, the incidence of adverse reactions of treatment group was reduced remarkably, indicating that pramipexole combined with ganglioside was more effective than dopasserzid alone in relieving symptoms and improving QOL in PD patients.

TNF-α has been shown to relieve depression’ symptoms. Although the pathogenesis of PD has not been uncovered, the environment and genetics may play very important roles in the cause of PD. During the study of PD, researchers found that the serum TNF-α level of PD patients was increased, which was also closely related to PD’ severity, indicating TNF-α as a potential biomarker for PD prognosis [29]. In this study, the serum TNF-α levels were significantly reduced after treatment, and the TNF-α level in the treatment group was obviously elevated relative to control group.

BDNF is a polypeptide hormone whose role is to repair damaged neurons, so the decline of BDNF is a signal of the occurrence of cognitive dysfunction [30], which shows that the level of BDNF can show the degree of cognitive dysfunction in patients. Studies have shown that serum cystatin C, as a cathepsin inhibitor, is closely related to many neurological diseases [31]. In this treatment, serum Cystatin C and CRP were significantly reduced, and in the statistics of the above two groups of data, the treatment group was decreased more significantly than the control group, and the BDNF was increased significantly, relative to the control group, which showing that pramipexole plays a very good role in regulating the serum content of PD. The possible reason is that the treatment group was given pramipexole combined with ganglioside treatment. Ganglioside has a strong antioxidant effect, which can effectively remove oxygen free radicals, reduce the level of malondialdehyde in the patient’s body, and increase the body’s anti-oxidant effect. The level of oxidase, which in turn achieves the effect of inhibiting the level of oxidative stress in the body [32], protects neurons and avoids free radicals from persecuting them. At the same time, gangliosides can increase the level of brain-derived neurotrophic factor, and it can effectively repair damaged neurons, thereby improving patients’ cognitive level [33].

The findings of this study demonstrate that the combination of pramipexole and ganglioside treatment for patients with Parkinson’s disease significantly improves clinical symptoms compared to the dopasrazide treatment group. This combination therapy can greatly reduce the occurrence of adverse reactions, improve motor function, and enhance patients’ quality of life. Pramipexole has a long half-life and can be rapidly absorbed after oral administration, allowing for sustained stimulation of the postsynaptic membrane and optimal therapeutic efficacy. Additionally, the synergistic effect of combining ganglioside with pramipexole can promote patient recovery while reducing the required dosage of drugs. This results in a lower incidence of adverse reactions, making the combination therapy a safer option for clinical application.

In summary, the combination of ganglioside and pramipexole has an ideal effect for PD treatment. It can effectively improve the patient’s condition, increase the level of BDNF, reduce the index level of cystatin C and CRP, and help eliminate the adverse effects of previous drugs. The clinical effect is obvious and it is worthy of promotion.

## Dodatak

### Conflict of interest statement

All the authors declare that they have no conflict of interest in this work.
